# N-terminal domain mutations of the spike protein are structurally implicated in epitope recognition in emerging SARS-CoV-2 strains

**DOI:** 10.1016/j.csbj.2021.10.004

**Published:** 2021-10-04

**Authors:** Apostolos Klinakis, Zoe Cournia, Theodoros Rampias

**Affiliations:** Biomedical Research Foundation Academy of Athens, 4 Soranou Efesiou, 115 27 Athens, Greece

**Keywords:** SARS-CoV-2, Virus evolution, N-terminal domain mutations, Immune escape

## Abstract

During the past two years, the world has been ravaged by a global pandemic caused by the severe acute respiratory syndrome coronavirus 2 (SARS-CoV-2). Acquired mutations in the SARS-CoV-2 genome affecting virus infectivity and/or immunogenicity have led to a number of novel strains with higher transmissibility compared to the original Wuhan strain. Mutations in the receptor binding domain (RBD) of the SARS-CoV-2 spike protein have been extensively studied in this context. However, mutations and deletions within the N-terminal domain (NTD) located adjacent to the RBD are less studied. Many of these are found within certain β sheet-linking loops, which are surprisingly long in SARS-CoV-2 in comparison to SARS-CoV and other related β coronaviruses. Here, we perform a structural and epidemiological study of novel strains carrying mutations and deletions within these loops. We identify short and long-distance interactions that stabilize the NTD loops and form a critical epitope that is essential for the recognition by a wide variety of neutralizing antibodies from convalescent plasma. Among the different mutations/deletions found in these loops, Ala 67 and Asp 80 mutations as well as His 69/Val 70 and Tyr 144 deletions have been identified in different fast-spreading strains. Similarly, deletions in amino acids 241–243 and 246–252 have been found to affect the network of NTD loops in strains with high transmissibility. Our structural findings provide insight regarding the role of these mutations/deletions in altering the epitope structure and thus affecting the immunoreactivity of the NTD region of spike protein.

## Graphical abstract

**Figure:**
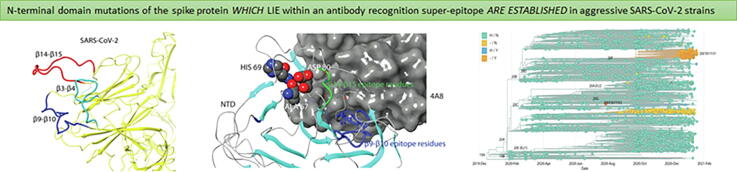

**Research in context**

*Evidence before the study*

The COVID-19 worldwide pandemic is already into the fourth wave, with novel highly aggressive strains expanding rapidly. Mutations in the RBD of the SARS-CoV-2 spike protein have been extensively studied in the context of higher infectability. However, accumulated evidence underlines the critical role of NTD mutations and deletions in the immunogenicity of the spike protein.

*Added value of this study*

In this study we find that certain loops within the N-terminal domain of the SARS-CoV-2 spike protein have evolutionary diverged in comparison to other beta-coronaviruses and particularly SARS-CoV. These highly flexible loops are in close proximity and contribute to various interactions that stabilize a surface-exposed tertiary structure. A super-epitope recognized by most neutralizing antibodies from convalescent plasma is formed and stabilized by specific amino acid residues within these loops, implying a critical role for epitope structure. For their length, these loops accumulate a disproportionably high number of mutations, driving SARS-CoV-2 evolution. Mutations and deletions affecting these amino acid residues are predicted to promote structural changes to the super-epitope region. Such mutations/deletions are common in fast-spreading SARS-CoV-2 strains associated with immune system escape.

*Implications of all the available evidence*

Together with available experimental data, our findings implicate mutations and deletions of NTD loop regions in immune evasion, and provide insights into the design of novel antibodies effective against these emerging strains.

## 1 Introduction

Coronaviruses (family: Coronaviridae) are RNA viruses with unusually large genome (∼30 Kb), which is of positive or plus-sense [1]. Coronaviruses are divided into four subtypes (α, β, γ and δ) and can infect various hosts from birds to mammals, and cause severe morbidity and mortality. They came under scrutiny in the past two decades due to recurrent incidents of widespread infection of humans. The most common human coronaviruses are the subtypes α 229E and NL63, and subtypes β HKU1 and OC43, which infect the respiratory tract of people throughout the globe causing common cold symptoms. More recently, β coronavirus strains of zoonotic (animal) origin have emerged as serious threats of human life: MERS-CoV (Middle East Respiratory Syndrome, MERS), SARS-CoV (Severe Acute Respiratory Syndrome, SARS) and SARS-CoV-2 (Coronavirus Disease 2019, COVID-19) [2]. Although the overall mortality of MERS and SARS-CoV is much higher, SARS-CoV-2, which emerged in the Wuhan province in China [3], has infected over 205,5 million people and caused close to 4.3 million deaths worldwide (https://coronavirus.jhu.edu/map.html).

SARS-CoV and SARS-CoV-2 share almost 80% sequence identity and both enter the host cells through interaction of the S (spike) protein with the angiotensin-converting enzyme 2 (ACE2) [4]. The S-protein shares 80% protein similarity between the two SARS strains and binds ACE2 with a similar affinity [5].

It is widely accepted that genetic variability and evolution within the positive strand RNA viruses is mainly driven by the low fidelity of RNA replication, as the RNA-dependent RNA polymerase (RdRP) is prone to high error rates [6]. Unlike other RNA viruses, where replication is primarily dependent on the RdRp, in coronaviruses, non-structural proteins (nsps) that include processivity factors (nsp7-8), a helicase (nsp13), a single-strand binding protein (nsp9), a proofreading exonuclease (nsp14) and other cofactors (e.g. nsp 10, nsp16) form a replication complex with the RdRp (that has proof-reading activity and corrects errors by the viral RdRp [7], [8]. As a result, coronaviruses are characterized by a 10-fold lower mutation rate compared to other RNA viruses. Despite the proofreading activity in RNA replication , the estimated mutation rate for SARS-CoV is 4x10^-4^ nucleotide substitutions/site/year [9], while for SARS-CoV-2 it is 1.12 × 10^−3^ mutations per site/year [10].

Recent studies have provided strong evidence that the SARS-CoV-2 spike protein NTD contains epitopes recognized by neutralizing antibodies produced by host adaptive immune response [11]. Moreover, it has been proposed that this region of the spike protein is dynamically involved in host cell surface adhesion, mediating interactions with glycan groups of the cellular glycoenvironment [12]. Therefore, tracking genetic variation in SARS-CoV-2 NTD is important for monitoring emerging strains with potentially higher capability for immune escape or with higher infectivity. To this direction, we investigated the evolution of NTD in β coronaviruses. By comparing the SARS-CoV and SARS-CoV-2 spike structures and analyzing the available mutation data for SARS-CoV-2, we discovered that specific NTD loop elements o which are evolutionary diverged in the SARS-CoV-2 clade, display high mutation rates and drive genetic variation. Interestingly, specific mutations and deletions in these loops are associated with an altered NTD structure, and affect an epitope region that is common among many different antibodies targeting the NTD region of the spike protein. The corresponding epidemiological data for these mutants revealed that specific NTD mutations and deletions have been positively selected over the last waves of COVID-19 pandemic.

## 2 Methods

### 2.1 Sequence alignment and secondary structure analysis

For sequence alignment of the NTD in SARS-related coronaviruses, spike protein sequences from SARS-CoV (PDB ID: 5X58), SARS-CoV-2(PDB ID: 6VSB), Bat-SL-CoVZC45 (ID: AVP78031), Bat-SL-CoVZXC21 (ID: AVP78042), Bat-SL-CoVBM48 (ID: NC_014470.1), Bat-SL-CoVHKU3 (ID: Q3LZX1.1) and Bat-SL-CoVRp3/2004 (ID: Q3I5J5.1) were used. Multiple sequence alignment was performed with the Clustal Omega program (version 1.2.4) [13]. For multiple sequence alignment visualization, the Jalview 2.11 software (http://www.jalview.org/) was used. To investigate the structural divergence of the SARS-CoV-2 NTD with respect to SARS-CoV, the secondary structure of the SARS-CoV NTD, as resolved by the cryo-EM structure (PDB ID: 5X4S), was compared to the cryo-EM structures of SARS-CoV-2 (PDB ID: 6VYB). Phylogenetic tree generation was performed using the ClustalW2 program.

### 2.2 Structural analysis and interloop interactions in SARS-CoV-2 NTD

The COVID-3D online platform (http://biosig.unimelb.edu.au/covid3d/), which implements the Arpeggio program for the calculation of interatomic interactions [14], was used for the visualization of residue interactions between the β3-β4, β9-β10 and β14-β15 loops. This analysis was performed on PDB files corresponding to the cryo-EM structure of SARS-CoV-2 at closed (PDB ID: 6VXX) and open (PDB ID: 6VSB) state after molecular modelling. According to the provided information by the COVID-3D platform, the structures were optimized by Maestro (https://www.schrodinger.com/maestro, Schrodinger suite, v. 2021-1) generating the PDB structures QHD43416_CLOSED (closed state) and QHD4316_ACE2_BOAT (open state) [15]. Surface electrostatic partial charges were generated with the Poisson-Boltzmann (APBS) method [16]. Visualization of SARS-CoV-2 spike protein trajectories was also performed using the BioExcel-CV19 platform designed to provide web-access to atomistic-MD trajectories for macromolecules involved in the COVID-19 disease (https://bioexcel-cv19.bsc.es/#/)

### 2.3 Mutation analysis

Global mutation data for SARS-CoV-2 NTD sequences were retrieved from the Global Initiative for Sharing All Influenza Data (GISAID) at https://www.gisaid.org/epiflu-applications/phylodynamics/. The frequency over time of SARS-CoV-2 strains harboring mutations on β3-β4, β9-β10 and β14-β15 loops was analyzed through GISAID from January 2020 to July 2021). Nextstrain and WHO nomenclature regarding the major SARS-CoV-2 clades was applied to our analysis for specific variants [23], [24].

### 2.4 Structural analysis of epitopes recognized by NTD targeting antibodies

Structural figures related to the epitopes recognized by NTD targeting antibodies were made using Maestro (Schrodinger suite, v. 2021–1) and the following cryo-EM data: 4A8 (PDB ID:7C2L), 5–24 (PDB ID:7L2F), 2–17 (PDB ID:7LQW) and 4–8 (PDB ID:7LQV).

### 2.5 Ethics

Ethical approval was unnecessary because this work is a meta-analysis of publicly available data.

### 2.6 Role of funders

The authors received no financial support for this research.

## 3 Results

### 3.1 Specific loop regions drive the evolutionary divergence of spike NTD protein in β coronaviruses

Sequencing of SARS-CoV-2 genomes from Wuhan patients in China and phylogenetic analysis of representative β coronavirus genomes (Genus: Betacoronavirus) revealed that the subgenus *Sarbecovirus* of the genus *Betacoronavirus* could be classified into three dinstict clades. Clade 1 consists of two SARS-CoV-related strains from Rhinolophus sp from Bulgaria (BM-48) and Kenya (BtKY72). Human SARS-CoV-2 sequences and two bat-SARS-like strains from eastern China (bat-SL-CoVZC45 and bat-SL-CoVZXC21) form the clade 2, while SARS-CoV strains from humans and bat SARS-like coronaviruses from southwestern China form the clade 3 [17].

**Fig. 1:**
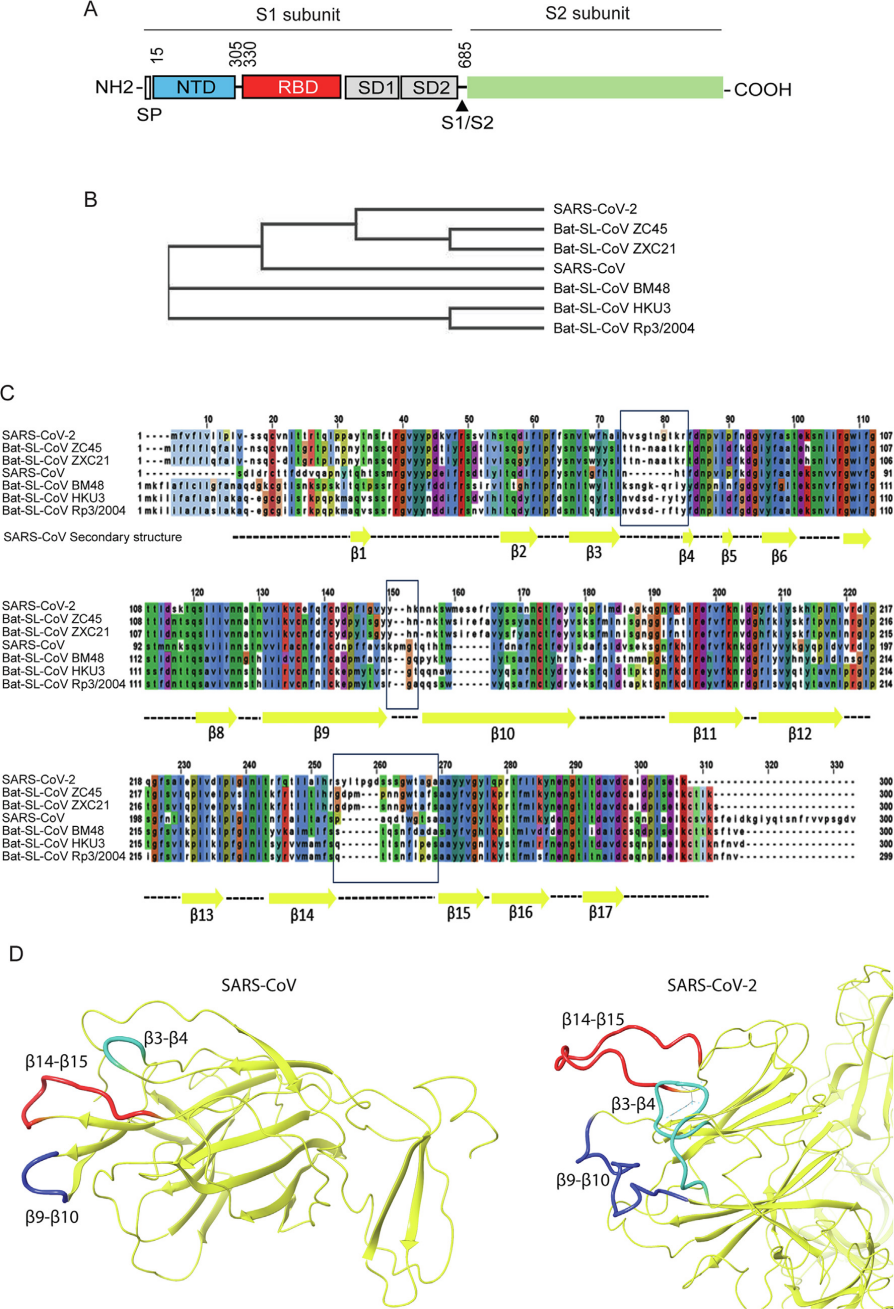
Sequence and structural properties of the SARS-CoV-2 spike protein NTD. (A) Schematic representation of the SARS-CoV-2 spike protein domains. (B) Evolutionary relationship between different β-coronaviruses based on protein alignment of the NTD spike sequence. (C) β-coronaviruses NTD sequence alignment. Secondary structure of the SARS-CoV NTD (PDB ID: 5X4S) has been mapped to alignment indicating structural differences among the different members. (D) Cryo-EM structures of the SARS-CoV spike protein (left; PDB ID: 5X4S) and SARS-CoV-2 (right; PDB ID: 6VYB) indicating the β3-β4 (turquoise color), β9-β10 (blue color) and β14-β15 (red color) loop regions. (For interpretation of the references to color in this figure legend, the reader is referred to the web version of this article.)

To investigate whether the protein alignment of the spike NTD in β coronavirus members displays a similar phylogenetic profile, we performed a comparative sequence analysis among the spike NTDs of representative β coronaviruses strains. In this direction, residues 15–305 of the SARS-CoV-2 spike protein (Fig. 1A) were aligned against the NTD sequences of SARS and members of clade 1 (BM48), clade 2 (BM48ZC45, ZXC21) and clade 3 (HKU3, Rp3) Bat-SARS like coronavirus strains. As Fig. 1B indicates, the protein alignment of these sequences showed that SARS-CoV-2 and the related bat-derived strains ZC45 and ZXC21 cluster together, while SARS-CoV and the bat SARS-like sequences cluster separately, demonstrating a similar phylogenetic pattern as the one acquired with the genomic sequences.

Integrating the secondary structure elements of SARS-CoV (as identified from the PDB: 5X4S structure) to this sequence alignment, we observed that overall, the NTD is well conserved within the majority of the β sheets and loops, with the exception of loops separating the β3-β4, β9-β10 and β14-β15 sheets (Fig. 1C). More specifically, SARS-CoV-2 β3-β4 and β14-β15 loops display an extended length compared to SARS-CoV. While an extended β3-β4 loop is also shared among all bat SARS-CoV members, β14-β15 loop extension is restricted only in SARS-Cov-2. To identify the structural differences in the NTD divergent regions between SARS-CoV-2 and SARS-CoV, the cryo-electron microscopy (Cryo-EM) structure of the SARS-CoV-2 spike protein [18] was compared against the SARS-CoV NTD crystal structure [19]. This structural comparison revealed that the length of β3-β4, β9-β10 and β14-β15 loops in SARS-CoV-2 has been evolutionarily extended with regard to SARS-CoV (Fig. 1D).

### 3.2 Interloop interactions and conformational stability of β3-β4, β9-β10 and β14-β15 domains

**Fig. 2:**
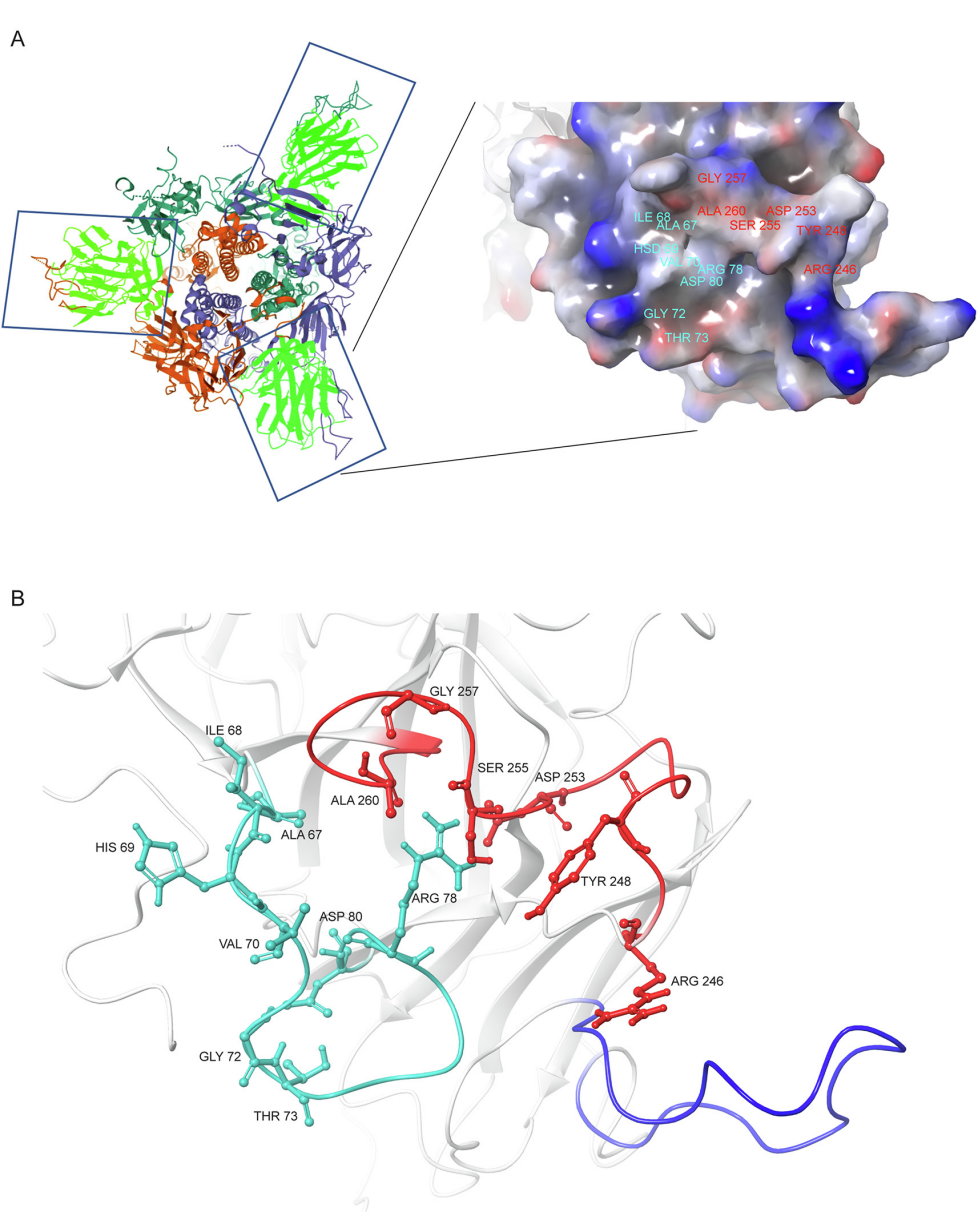
Structural features of β3-β4, β9-β10 and β14-β15 loop regions. (A) The location of the β3-β4, β9-β10 and β14-β15 loops within the trimeric S protein complex. Boxes indicate the N-terminus domain of the spike protein (in light green), while the loops are colored differently in each monomer. Surface area covered by β3-β4, β9-β10 and β14-β15 loops, and projected positive (blue color) and negative (red color) electrostatic potential. (C) Structural conformation of β3-β4 (turquoise), β14-β15 (red) and, β9-β10 (blue) loops. (For interpretation of the references to color in this figure legend, the reader is referred to the web version of this article.)

According to the cryo-EM structure of the SARS-CoV-2 trimeric spike complex and its proposed conformational states [18], [20], these loops are highly flexible and exposed on the outer surface of trimeric spike complex, away from the RBD. The protein surface corresponding to these interacting loops is hydrophilic and possesses a positive potential, due to the presence of several charged/hydrophilic amino acids (Fig. 2A). Notably, the β3-β4 and β14-β15 loops (amino acids 62–80 and 242–263, respectively) are in close proximity, stabilized by electrostatic interactions between amino acids in both loops (Fig. 2B).

**Fig. 3:**
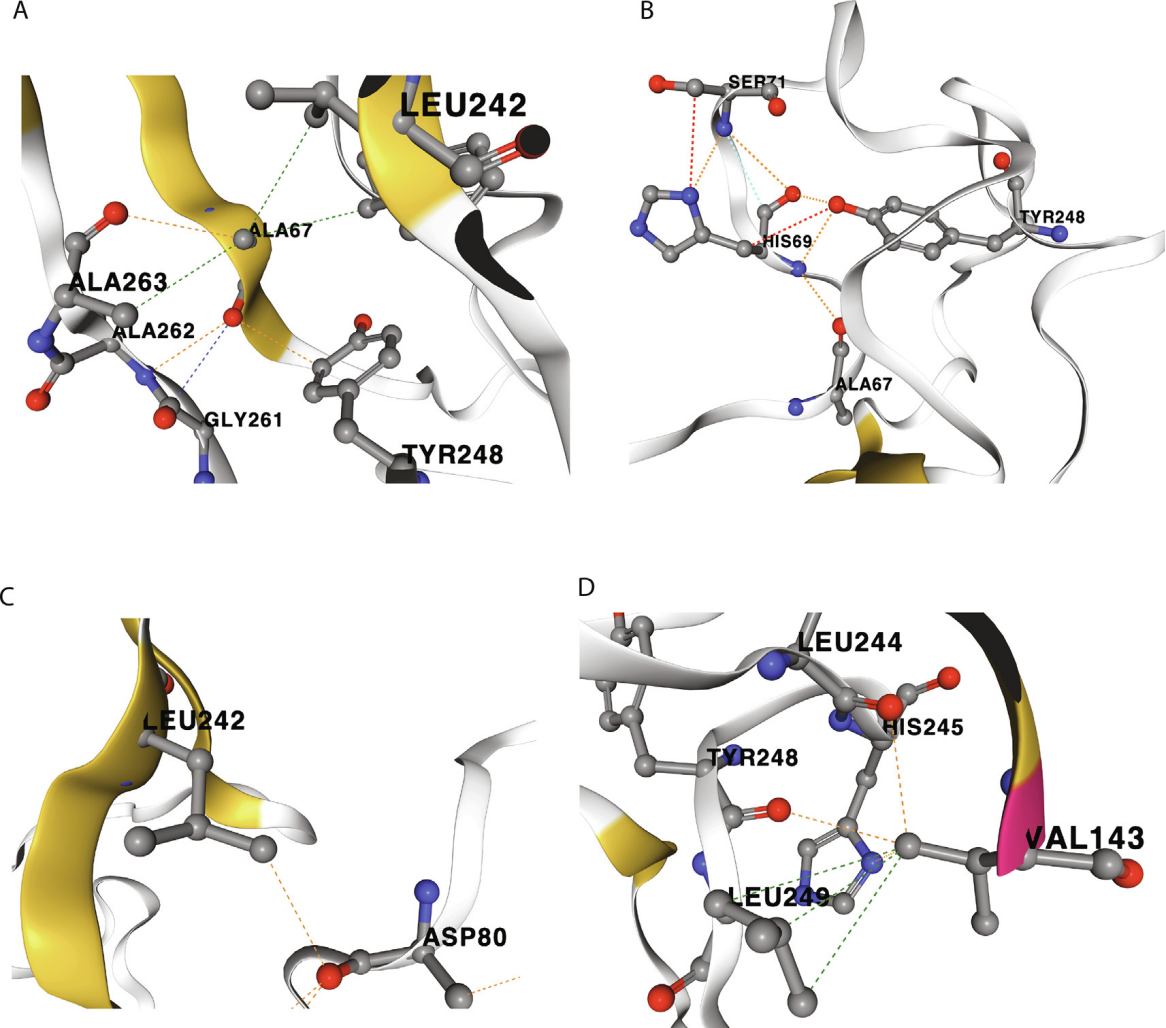
Interloop interactions. (A) Interaction of Ala 67 (β3-β4 loop) with Leu 242 and Tyr 248 residues (β14-β15 loop) as well as adjacent Gly 261, Ala 262 and Ala 263. (B) Interaction of His 69 (β3-β4 loop) with Tyr 248 (β14-β15 loop). (C) Interaction of Asp 80 (β3-β4 loop) with Leu 242 (β14-β15 loop). (D) Interaction of Val 143 (β9-β10 loop) with Leu 244, Tyr 248, Leu 249 and His 245 residues (β14-β15 loop). Dashed lines indicate direct interactions (red: hydrogen bonding, Blue: carbonyl-carbonyl, yellow: ionic, orange: polar, green: hydrophobic, light green: aromatic, light blue: Van der Waals). Modelling and visualization were performed within the COVID-3D Biosig portal. (For interpretation of the references to color in this figure legend, the reader is referred to the web version of this article.)

Our structural analysis revealed that a network of electrostatic and hydrophobic interactions between several residues of loops β14-β15 and β3-β4 mediate interloop communication. More specifically, the β3-β4 loop amino acid residues Ala 67 and His 69 interact with Leu 242, Tyr 248, Gly 261, Ala 262, Ala 263 and Tyr 265 amino acids of the β14-β15 loop (see representative interactions in Fig. 3A, B). Additionally, Asp 80, also in the β3-β4 loop, interacts with Leu 242 in the β14-β15 loop further stabilizing this tertiary structure(Fig. 3C). Finally, Val 143, in the β9-β10 loop, interacts with multiple amino acids of the β14-β15 loop (Leu 244, His 245, Tyr 248 and Leu 249), further stabilizing interloop interaction (Fig. 3D).

**Fig. 4:**
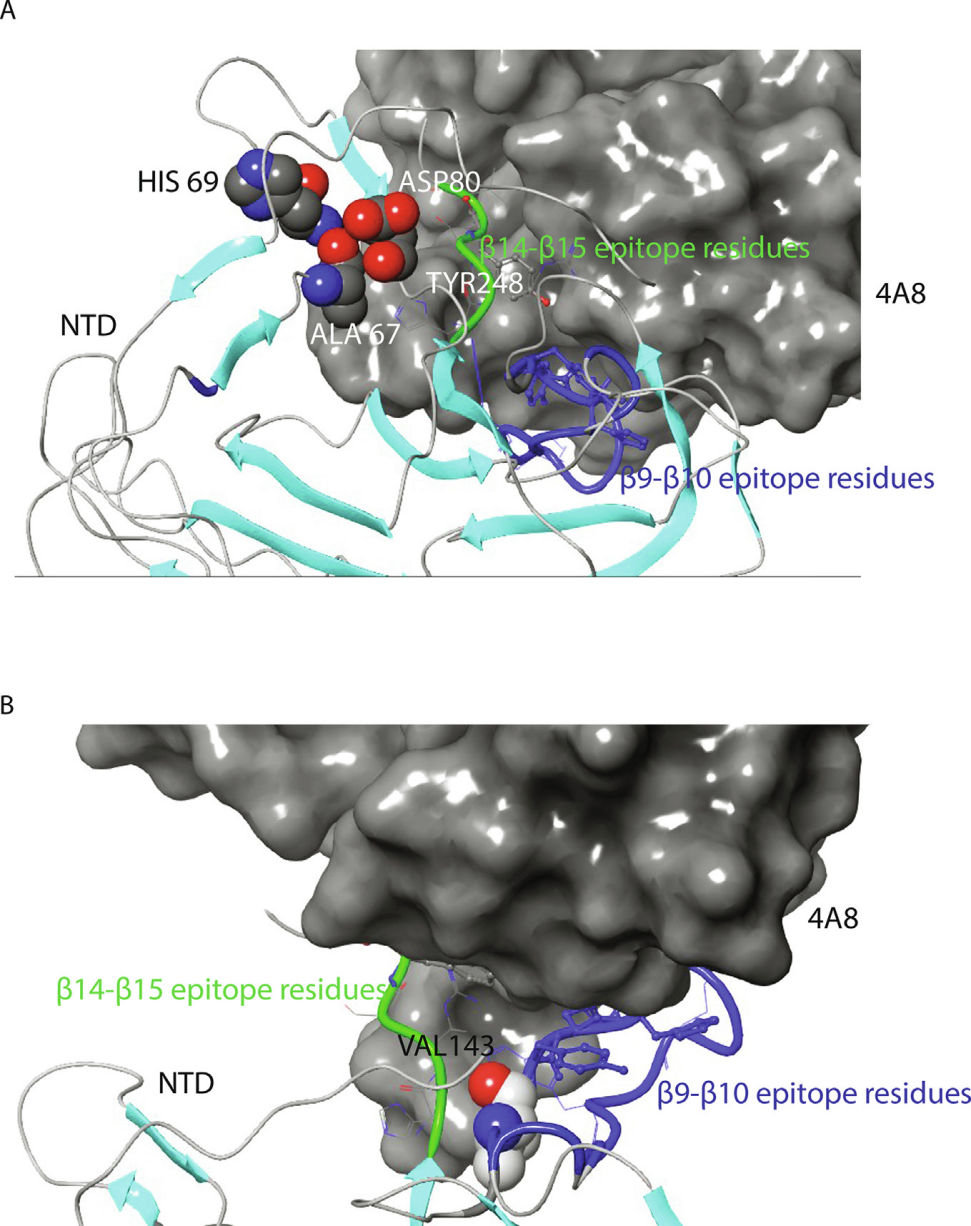
Relative location of β3-β4, β9-β10 and β14-β15 loop regions in the complex of the 4A8 antibody with the SARS-CoV-2 NTD. (A) Position of Ala 67, His 69, Asp 80 (β3-β4 loop) and Tyr 248 (β14-β15 loop). (B) Position of Val 143 (β9-β10 loop). Blue color indicates the β9-β10 loop residues 141–158. Structures are based on PDB 7C2L (Cryo-EM structure of 4A8:SARS-CoV-2 S complex). (For interpretation of the references to color in this figure legend, the reader is referred to the web version of this article.)

Interestingly, 4A8, one of the best characterized neutralizing antibodies [11], was the first member of NTD targeting antibodies to recognize a discontinuous epitope (ID: 1087268, Immune Database and Analysis Resource-IEDB) encompassing β9-β10 and β14-β15 amino acids (Tyr 144, Tyr 145, His 146, Lys 147, Lys 150, Trp 152, His 245, Arg 246, Ser 247, Tyr 248, Leu 249). These amino acids are positioned in the recognition interface within 4 Å distance from the antibody. As mentioned above, several of the epitope amino acids participate in an extensive network of interactions with residues Ala 67, His 69 and Asp 80 (Fig. 4A), and Val 143 (Fig. 4B), suggesting that these interactions are important for antibody recognition.

**Fig. 5:**
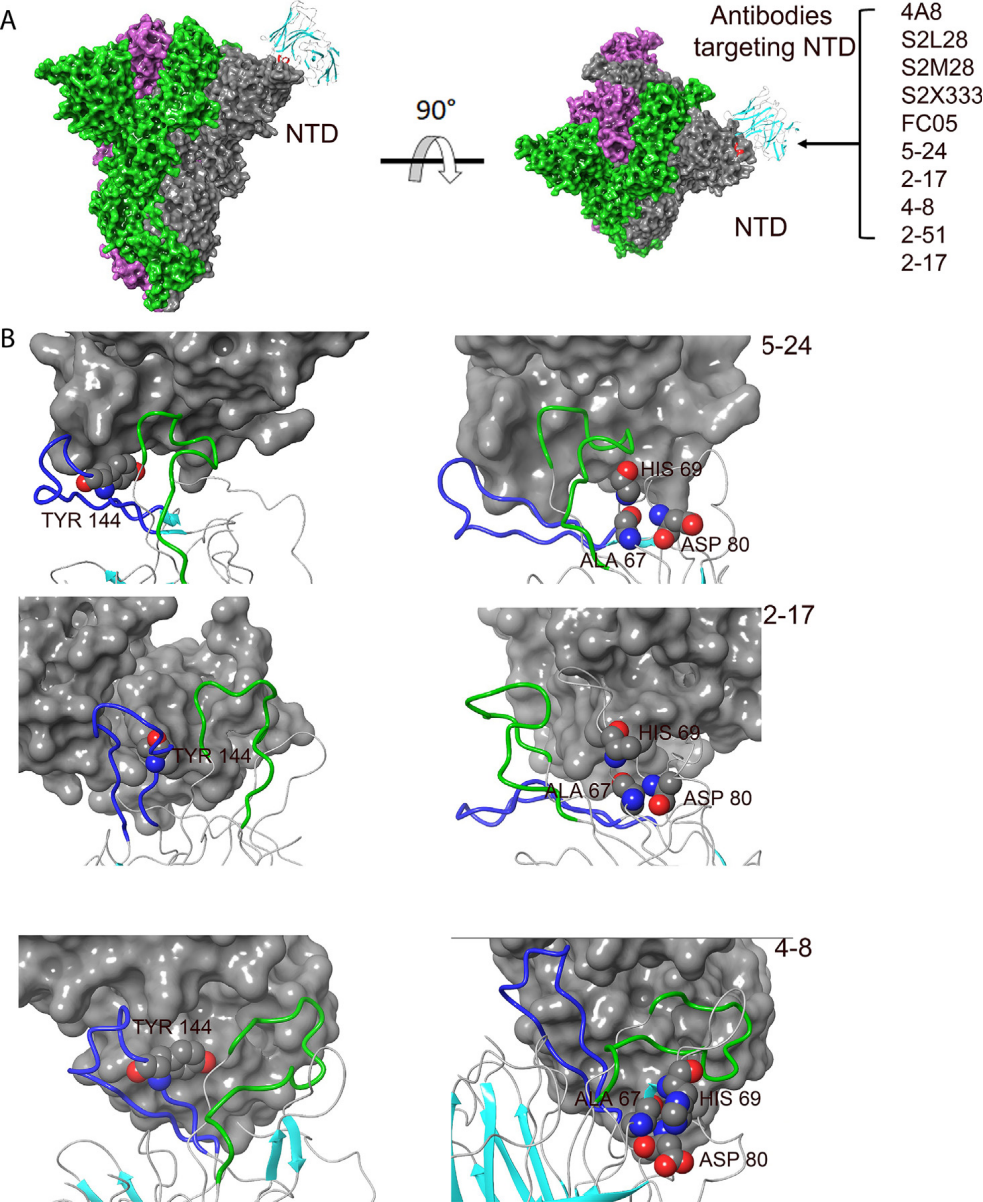
Interaction between NTD-targeting antibodies and loops β3-β4, β9-β10 and β14-β15 of the SARS-CoV-2 spike protein. (A) Structural presentation of trimeric spike protein binding by characterized NTD-targeting antibodies. (B) Cryo-EM structures of the epitope recognized by the 5–24 (PDB ID:7L2F), 2–17 (PDB ID:7LQW) and 4–8 (PDB ID:7LQV) antibodies. Position of Ala 67, His 69, Asp 80 (β3-β4 loop) and Tyr 144 (β9-β10 loop) are shown. Green color indicates loop β14-β15; blue color indicates loop β9-β10. (For interpretation of the references to color in this figure legend, the reader is referred to the web version of this article.)

Recent studies have highlighted the immunogenic properties of NTD and, besides 4A8, a wide panel of neutralizing antibodies have been identified to recognize a NTD supersite [21]. Our structural analysis of epitopes recognized by COV57 [22], S2L28, S2M28, S2X333 [23], 2–17, 5–24, 4–8 and 2–51 [24] antibodies revealed that β3-β4, β9-β10 and β14-β15 loops have an important role in the formation of this universal epitope (Fig. 5A, B). Therefore, mutations or deletions that affect amino acids in these loops are expected to remodel this epitope and alter the binding affinity of these antibodies. Masking of exposed amino acid residues by glycosylation has been described as a general mechanism of viral immune evasion [25]. It is thus important to mention that two glycosylation sites have been identified in loops β3-β4 and β14-β15, at residues Asn 74 and Asn 149 [26].

### 3.3 Amino acid variability on β3-β4, β9-β10 and β14-β15 loops modulates the evolutionary dynamics of the spike protein NTD region in SARS-COV-2 strains

**Fig. 6:**
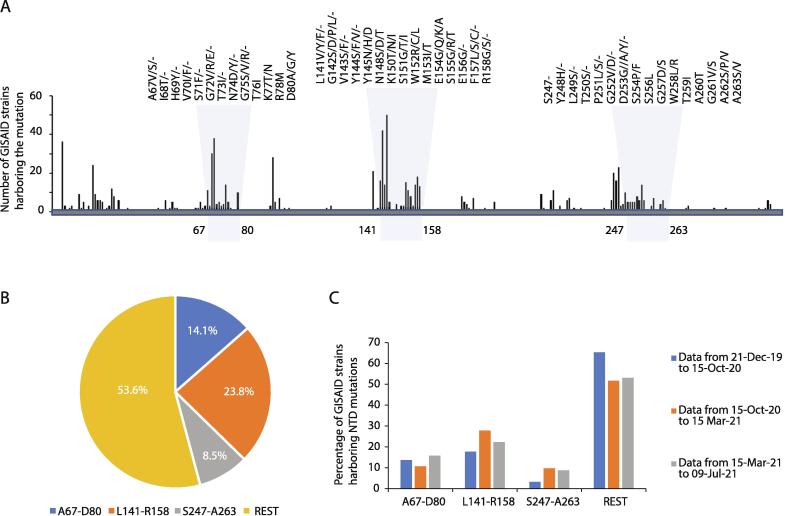
Mutations associated with the N-terminus domain of SARS-CoV-2 spike protein. (A) Distribution and relative frequency of mutations in the N-terminus amino-acid sequence of SARS-CoV-2 spike protein. Mutation frequency has been calculated based on the number of GISAID strains harboring nonsynonymous mutations/deletions in each amino acid position. (B) Global frequency of mutations in β3-β4, β9-β10 and β14-β15 loop regions calculated as percentages of the total mutations in the SARS-CoV-2 NTD region (aa 1–350). (C) Relative frequency of mutation for indicated loops during early, middle and recent phase of the COVID-19 pandemic.

To investigate the distribution of identified SARS-CoV-2 mutations in the NTD secondary structure, mutation data from 2,022,459 high-quality genomic sequences were analyzed through the GISAID SARS-CoV-2 database (https://www.gisaid.org/) as of July 9th, 2021, and aligned to the 1–350 aa sequence of the SARS-CoV-2 spike protein (YP_009724390). We observed that approximately 46.4% of identified NTD non-synonymous mutations in different SARS-CoV-2 strains are found within the β3-β4, β9-β10 and β14-β15 loop regions, indicating a higher degree of variation in these elements compared to other NTD secondary structure elements (Fig. 6A-C). Since the β3-β4, β9-β10 and β14-β15 loops are positioned away from the NTD core and the interaction surface with the RBD, mutations therein may be under reduced selective pressure. On the other hand, most of the identified loop variants affect the solvent-accessible surface of the spike protein, and these mutations can potentially affect the dynamics of intermolecular interactions with sugars or antibodies. This is particularly important for RNA viruses, as it is known that high mutation rate on their proteins is associated with escape from host immune response, higher virulence and altered tissue tropism [27], [28], [29]. Interestingly, mutations in the β3-β4 and β9-β10 loops display a higher frequency compared to the β14-β15 one, suggesting a more dynamic role of mutations in these two regions during SARS-CoV-2 evolution.

### 3.4 Specific mutations within the β3-β4 and β9-β10 loops can lead to conformational changes

Since we observed that mutations within the β3-β4 and β9-β10 loops are quite frequent, we sought to investigate whether specific mutations in these regions can modulate the conformational stability of NTD. As mentioned above, our analysis on the 3D structure of the spike protein revealed that Ala 67, Asp 80 and Val 143 residues maintain a rigid network of interloop interactions. Therefore, mutations in these amino acids may alter the stability of β3-β4/β14-β15 interactions. Indeed, substitution of Ala by Val at position 67 (A67V) is associated with the establishment of novel hydrophobic interactions with Ile 100, Phe 79 and Ala 263 residues (Supplementary Fig. 1A). Moreover, a number of interactions of Asp 80 (Supplementary Fig. 1B) are lost upon mutation to Tyr (Supplementary Fig. 1C) or Ala (Supplementary Fig. 1D). Specifically, D80Y abolishes interactions with His 66, while D80A does not interact with Phe 65, His 66 and Pro 82. The association of these mutants with a rewired network of interactions may induce conformational changes. On the other hand, substitution of Val by Phe at position 143 (V143F) is predicted to result in loss of aromatic and hydrophobic interactions with Leu 244, His 245 and Leu 249.

### 3.5 Epidemiological data provide evidence that specific mutations and deletions within the β3-β4, β9-β10 and β14-β15 loops have undergone a positive selection during SARS-CoV-2 evolution

The high mutation rate of viruses provides unique opportunities for natural selection of strains based on greater stability, higher transmission rates and immune escape. As a result, certain variants show increasing representation within the population, through a positive selection [30].

As of June 2021, Nextstrain has identified 13 major clades (19A–B, 20A–20J and 21A). During SARS-CoV-2 evolution, different mutated strains emerged within these clades, displaying high transmissibility, and thus affecting COVID-19 epidemiology. A global monitoring of these strains, also known as variants of concern (VOCs) and variants of interest (VOIs), has been established as a response to COVID-19 pandemic, and a single Greek letter naming scheme has been adopted by the World Health Organization (WHO) for easier labelling. By July 15, 2021, a high global distribution of Alpha (Pango lineage designation B.1.1.7), Beta (B.1.351), Gamma (B.1.1.28.1) and Delta (B.1.617.2) VOCs has been reported (WHO epidemiological updates: https://www.who.int/en/activities/tracking-SARS-CoV-2-variants/). According to the released reports of WHO epidemiological data, transmissibility in Alpha, Beta, Gamma and Delta strains has increased by 29%, 25%, 38% and 97%, respectively, compared to the original Wuhan strain [31]. Similarly, multiple VOIs such as Eta (B.1.525), Iota (B.1.526), Kappa (B.1.617.1) and Lambda (C.37) have been identified to represent an emerging risk, due to their increasing prevalence. Epidemiological evidence also exists that genomic properties of the B.1.1.318, B1.1.375 and B.1.1.616 strains have a significant impact on transmissibility, and these strains have been characterized as variants under monitoring.

**Fig. 7:**
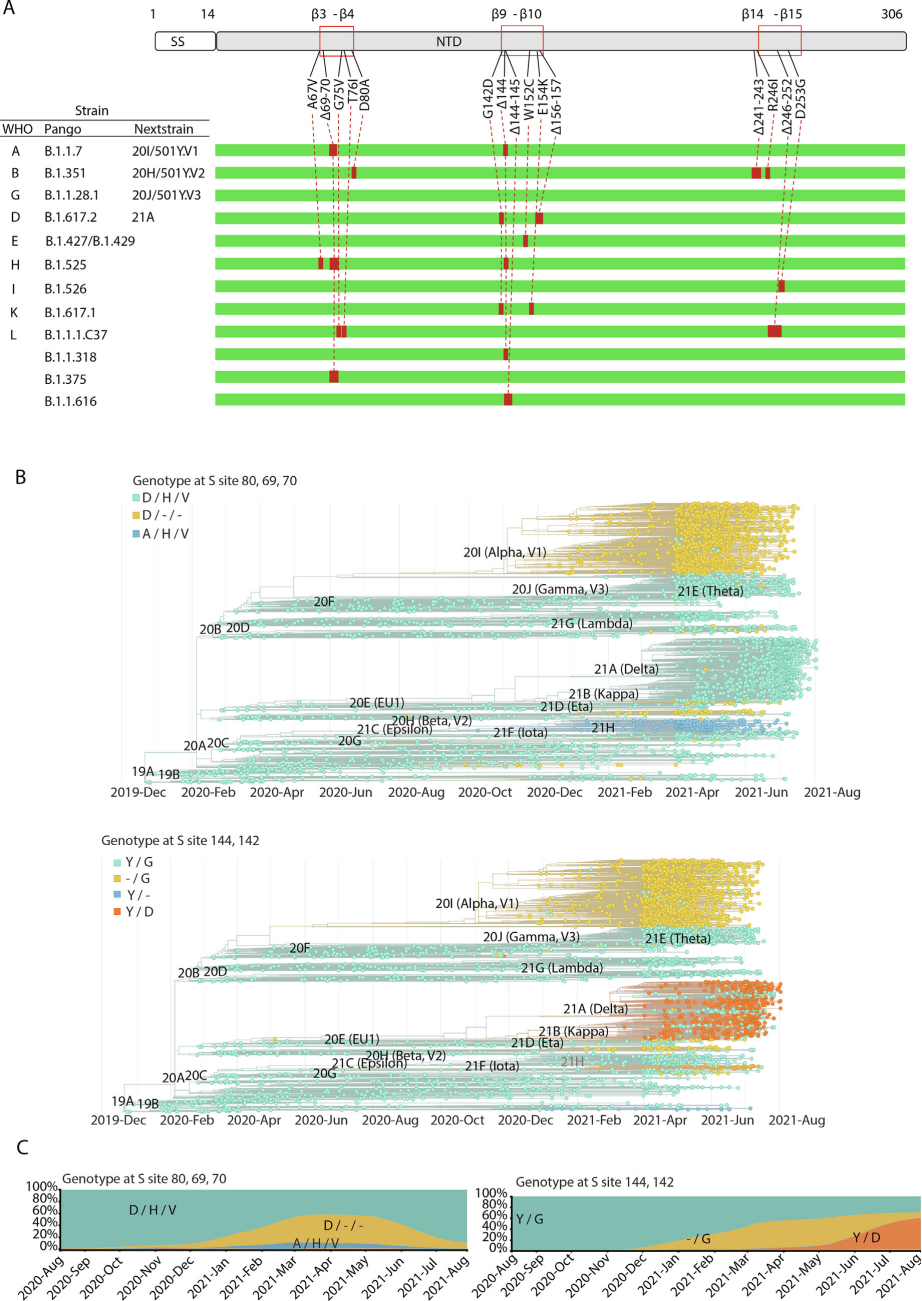
Epidemiology of mutations and deletions in loops β3-β4, β9-β10 and β14-β15 of the NTD region. (A) Distribution among VOCs, VOIs and strains under monitoring. (B) Phylogeny of major SARS-CoV-2 clades with mutations/deletions of amino acid residues Asp 80, His 69, Val 70 (top), and Gly 142 and Tyr 144 (bottom). (C) Area plot indicating the prevalence of each genotype globally. The Y axis indicates frequency (%) and the x axis indicates the timeline. All mutations refer to the spike protein.

The association of the reported VOCs/VOIs and the currently designated variants under monitoring B.1.1.318, B1.1.375 and B.1.1.616 with NTD mutations/deletions in β3-β4, β9-β10 and β14-β15 loops revealed that besides Gamma strain, all the other highly aggressive strains are characterized by multiple alterations on these three loop regions that form the universal NTD epitope (Fig. 7A). Notably, deletions are distributed among all loop regions with a specific enrichment for Δ69-70 and Δ144 in β3-β4 and β9-β10 loops respectively, which are present in several different VOCs/VOIs (Alpha, Eta, B.1.375, B.1.1.616). Moreover, a co-occurrence of Δ69-70 with Δ144 exists in Alpha and Eta strains. As a result, the frequency of these two deletions in SARS-COV-2 genotyped sequences from patients has become very high in recent months (Fig. 7B, C). Besides Δ144, the β9-β10 loop, which has a critical role in the formation of the NTD antigenic supersite, seems to display different patterns of deletions in aggressive strains, as Δ144-145 and Δ156-157 have also been identified in the B.1.1.616 and Delta strains. Interestingly, deletions in the β14-β15 loop have been found so far only in the Lambda strain harboring the Δ246-252 deletion (Supplementary Fig. 2). Notably, Δ241-243, which is present in the Beta strain, affects a region adjacent to the β14-β15 loop, while its impact on the supersite formation has not been studied yet.

In VOCs/VOIs and variants under monitoring, the above pattern of deletions coexists with specific missense mutations. More specifically, mutations in the β3-β4 loop that are associated with the genetic properties of these strains include A67V, G75V, T76I and D80A. On the other hand, G142D, W152C and E154K within the β9-β10, and R246I and D253G in the β14-β15 loop, are the missense mutations identified in the other two loops (Fig. 7A). Despite the mutation/deletion overlap among strains, the different combinations of the genetic variations generate complex virus genotypes (Fig. 7B, C and Supplementary Fig. 2), while the functional implications of these combinations are difficult to assess.

These acquired alterations are expected to modify the network of interloop and intraloop interactions, and thus to induce structural remodeling of the NTD antigenic supersite. In fact, deletions within the above loops have been proposed to decrease the binding and neutralization potency of COVID-19 patient convalescent sera or monoclonal antibodies (mAbs). For this reason, it is considered that these deletions facilitate immune escape and are thus positively selected [32], [33]. On the other hand, recent studies have highlighted the role of specific missense mutations in structural changes of the NTD antigenic supersite. Functional data have also correlated these changes with immune escape. For instance, the W152C mutation introduces a free cysteine that can form new disulfide bonds. In the Epsilon (B.1.427/B.1.429) strain, an alternative disulfide bond between C136 and W152C is proposed to drive NTD conformational changes that lead to immune escape from NTD targeting neutralizing antibodies [34]. Moreover, G142D has also been shown to alter the binding of NTD targeting antibodies [23]. Notably, Ala 67 and Asp 80 residues in the β3-β4 loop, which we predict to maintain interloop interactions in the NTD supersite, are mutated in Eta (A67V) and Beta (D80A) strains. In line with our analysis, an NTD remodeling induced by D80A has also been proposed recently [35].

## 4 Discussion

SARS-CoV-2 is genetically related to SARS-CoV, a deadly coronavirus that emerged in late 2002 and caused an outbreak of severe acute respiratory syndrome. SARS-CoV was highly lethal but after intense public health measures, was eradicated in 2003 [36]. The new coronavirus SARS-CoV-2 is less deadly but far more transmissible [37]. Moreover, while SARS‐CoV appears to infect pneumocytes and enterocytes of the small intestine [38], SARS-CoV-2 can infect multiple organs such as the intestine, liver, kidney and blood vessels [39], [40].

To identify divergent structure elements on the SARS-CoV-2 spike protein NTD that could potentially modulate interactions with the host, we performed a comparative sequence and structural analysis on SARS-CoV and SARS-CoV-2 NTDs. As expected, NTD sequences of SARS-CoV and SARS-CoV-2 are highly similar. The most striking difference is the length of loops β3-β4, β9-β10 and β14-β15 which are significantly longer in the SARS-CoV-2 and in certain bat coronaviruses with genomes closely related to SARS-CoV-2, indicating that the structural evolution of these elements is characteristic for the SARS-CoV-2 clade identity. The low degree of homology between sequences corresponding to the respective loops from bat and human β-coronaviruses, suggests that amino acid variations in these elements have a major impact on divergence of spike proteins within the SARS-CoV-2 clade. Although the cryo-EM data suggest that these divergent loop regions are part of a highly flexible NTD region, our molecular modeling data indicate that a network of electrostatic and hydrophobic interactions between several residues of β3-β4 and β9-β10 loops with residues of the β14-β15 loop mediate an interloop communication that provides a relative stability. Residues Ala 67, His 69 and Asp 80 in β3-β4 and Val 143 in the β9-β10 loop were identified to play an important role in these interactions.

It is well accepted that antibodies targeting the RBD confer significantly to the neutralizing activity of convalescent sera [41], [42]. In a recent study however, Voss and colleagues analyzed the proteomic profile of IgGs in convalescent sera and demonstrated that the response is directed predominantly (>80%) against epitopes residing outside the RBD, and include the NTD region and the S2 domain [43]. The same study also reported that anti-NTD antibodies contribute critically to neutralization and their protection is related to their relative levels in plasma.

To this direction, numerous immunological studies have demonstrated that NTD-targeting antibodies elicited in COVID-19 patients are effective neutralizing antibodies (nAbs). In a recent study by Liu and colleagues, almost half of the neutralizing Abs isolated from plasma derived from 40 COVID-19 patients exhibited binding to NTD but not to RBD [24]. Neutralizing antibodies 4A8 [11], COV57 [22], 2–17, 5–24, 4–8 [24] and possibly others [44] target the exposed NTD surface of the SARS-CoV-2 spike protein. Moreover, while RBD-targeting neutralizing antibodies recognize distinct epitopes, various neutralizing antibodies against the NTD target a common site comprising primarily loops β9-β10 and β14-β15 (N3 and N5 according to Chi et al) [21]. It has been postulated that this region is highly immunogenic in part because it is glycan free, which allows epitope recognition. Moreover, the high flexibility of the loops allows the peptide to assume multiple conformations accommodating recognition by several antibodies. The neutralizing activity of NTD antibodies relies on hindrance, which prevents the spike protein from binding to the ACE-2 receptor [21].

It is well accepted that the conformation of epitopes is essential for the neutralizing activity of antibody responses against SARS-CoV [45], whereas specific mutants can escape the neutralizing activity of certain antibodies [46]. Since conformation changes in the β3-β4, β9-β10 and β14-β15 loops could possibly alter NTD antibody recognition, we analyzed the GISAID mutation data in order to study the sequence variation profile of SARS-CoV-2 divergent loops. Our analysis revealed a disproportionate high rate of mutations in these loops, indicating a dynamic role in spike sequence divergence and evolution within SARS-CoV-2 clade that could enhance immune escape

Based on our molecular modeling of the spike 3D structure, Ala 67, Asp 80 and Val 143 residues maintain a rigid network of interactions. In this context, A67V, D80Y, D80A and V143F variants are predicted to rewire the network of these interactions, and to either promote the establishment of new hydrophobic interconnections (A67V) or induce a loss of intraloop hydrogen bonds (D80Y, D80A) and interloop hydrophobic interactions (V143F). This might hinder NTD recognition by neutralizing antibodies from convalescent plasma. In this context, recent studies revealed that in frame deletions of NTD amino acid sequence in SARS-CoV-2 strains that affect β3-β4 (Δ69-70), β9-β10 (Δ141-144, Δ146) and β14-β15 loops (Δ243-244) are associated with immune escape in patients [32].

In order to identify whether SARS-CoV-2 strains harboring mutations in NTD loops are associated with greater prevalence, we investigated Nextstrain data with a focus on WHO reported VOCs (Alpha, Beta, Gamma, Delta), VOIs (Eta, Iota, Kappa and Lambda) and strains under monitoring (B.1.1.318, B1.1.375 and B.1.1.616) that are characterized by high transmissibility. Notably, for many of these prevalent strains, recent functional studies have established a link between their high transmissibility and their ability to escape from RBD and NTD targeting neutralizing antibodies. Regarding the escape from NTD targeting antibodies, strong experimental evidence exists for Alpha and Beta [35], [47], [48], [49], as well as for Delta [50], [51] and Epsilon [34] strains.

This analysis revealed that with the exception of the Gamma strain, all other highly aggressive strains harbor multiple mutations and deletions in β3-β4, β9-β410 and β14-β15 loops. The high prevalence of deletions in these loop regions, may indicate that deletions might be subjected to a stronger selection for antigenic drift than missense mutations.

The structural analysis of complexes between neutralizing antibodies and the NTD suggests that the β9-β410 and β14-β15 loops are closer to the antibody binding surface than the β3-β4 loop, which is not directly involved in interactions with antibody residues. However, our structural analysis revealed an important role for the β3-β4 residues Ala 67, His 69 and Asp 80 in interloop interactions that stabilize the β14-β15 loop. Since β14-β15 loop is a critical part of the NTD antigenic supersite, β3-β4 loop mutations may have a critical role in immune escape. Interestingly, Ala 67 and Asp 80 are mutated in Eta (A67V) and Beta (D80A) strains, while His 69 is deleted (Δ69-70) in Alpha, Eta and B.1.375 strains. Our analysis also indicated that Val 143 interacts with multiple amino acids of the β14-β15 loop (Leu 244, His 245, Tyr 248 and Leu 249), and thus stabilizes it in a folding critical for antigenicity. Although, Val 143 is not mutated within the group of VOCs/VOIs and other variants under monitoring, mutations of neighboring residues Gly 142 and Tyr 144 are present in Alpha (Δ144), Eta (Δ144), Delta (G142D), B.1.1616 (Δ144-145) and B.1.617.1 (G142D) strains.

We acknowledge that all computational data presented here merit experimental validation. Nevertheless, we have uncovered important aspects regarding virus interaction with the host immune system. Our findings could facilitate the generation of monoclonal antibodies and vaccines with better profile against novel fast-spreading SARS-CoV-2 variants.

## CRediT authorship contribution statement

**Apostolos Klinakis:** Conceptualization, Writing - original draft, Writing - review & editing, Visualization. **Zoe Cournia:** Software, Data curation, Visualization, Writing - original draft. **Theodoros Rampias:** Conceptualization, Software, Data curation, Writing - original draft, Writing - review & editing, Visualization.

## Declaration of Competing Interest

The authors declare that they have no known competing financial interests or personal relationships that could have appeared to influence the work reported in this paper.
