# Supplementary material for: N-terminal domain mutations of the spike protein are structurally implicated in epitope recognition in emerging SARS-CoV-2 strains
Source: Comput Struct Biotechnol J. 2021 Oct 4;19:5556–67. doi: 10.1016/j.csbj.2021.10.004 (PMC8489513; doi:10.1016/j.csbj.2021.10.004)
Supplement: Supplementary data 1 [file mmc1.docx]

**SUPPLEMENTAL INFORMATION**

N-terminal domain mutations of the spike protein are structurally implicated in epitope recognition in emerging SARS-CoV-2 strains

*Apostolos Klinakis^1,*^, Zoe Cournia^1^ and Theodoros Rampias^1,*^*

**^1^ Biomedical Research Foundation Academy of Athens, Athens, Greece.**

***Correspondence:** [**aklinakis@bioacademy.gr**](mailto:aklinakis@bioacademy.gr)**;** [**trampias@bioacademy.gr**](mailto:trampias@bioacademy.gr)

**Supplementary Figure 1. Hydrophobic and aromatic interactions in NTD mutants.** (A) Predicted intraloop and interloop interactions of Val at position 67 mutated from Ala (A67V). (B) Asp 80 interactions with Phe 65, His 66, Pro 82, Leu 242 and Tyr 265. (C) Loss of interactions with His 66 when Asp 80 is mutated to Tyr (D80Y). (D) Loss of intraloop interactions upon mutation to Ala (D80A). Dashed lines indicate direct interactions (blue: carbonyl; green: hydrophobic, light green: aromatic; red: hydrogen; orange: polar). Modelling and visualization was performed within the COVID-3D Biosig portal.

**Supplementary Figure 2. Phylogeny of deletions in loop β14-β15.** Phylogenetic tree indicating the time of appearance of GISAID strains harboring deletions Δ241-241 and Δ246-252. (B) Area plot indicating the prevalence of each genotype globally. The Y axis indicates frequency (%) and the x axis the timeline. All mutations refer to the spike protein.
